# A new resource for identifying and assessing the impacts of research

**DOI:** 10.1186/s12916-015-0364-x

**Published:** 2015-06-24

**Authors:** Saba Hinrichs, Jonathan Grant

**Affiliations:** The Policy Institute at King’s College London, 1st Floor, Virginia Woolf Building, 22 Kingsway, London, WC2B 6LE UK

**Keywords:** Impact, Health gains, QALY, Research Excellence Framework

## Abstract

The impact case studies submitted by UK Higher Education Institutions to the Research Excellence Framework (REF) in 2014 provide a rich resource of text describing impact beyond academia and across all disciplines. Using text mining techniques and qualitative assessment, the 6,679 non-redacted case studies submitted were analysed and the impact described was found to be multidisciplinary, multi-impactful, and multinational. By digging deeper into the data, the health gains from health research in terms of Quality Adjusted Life Years was also estimated. Similar analyses are possible using these case studies, but will require the data to be ‘re-purposed’ from the original intention (i.e., for assessment purposes) for robust analysis.

## Introduction

The Research Excellence Framework (REF) is a new system for assessing the quality of research in UK Higher Education Institutions (HEIs). The higher education funding bodies use the assessment outcomes to inform the selective allocation of their research funding. REF 2014 replaced the Research Assessment Exercise, which has occurred on a (near) quinquennial basis since 1986. Under this new system, HEIs are assessed on three criteria: outputs, e.g., publications (weighted at 65 % of the overall score), non-academic impact in the form of a set of impact case studies (20 %), and research environment (15 %).

The allocation of research funding based on non-academic impact is relatively new, with the REF being the first example of its application across a research system [1]. In 2006, a pilot exercise was carried out during the development of the Australian national Research Quality Framework which would have introduced impact assessment into their national research assessment exercise, but this was dropped with the change of government in 2007 [2]. In the UK, following a pilot exercise [3], the higher education funding bodies concluded that peer review of research impact case studies was a workable approach and it was decided that REF will assess universities on the basis of the quality of research outputs, the vitality of the research environment, and the wider impact of research.

An impact case study is a short four-page document consisting of five sections: i) summary of impact, ii) a description of the underpinning research, iii) references to that research, iv) details of the impact, and v) sources to corroborate the impact. These case studies are now available in an online searchable database [4].

A total of 154 HEIs made REF submissions, with the number of case studies per submission ranging from 2 to 260 – providing a unique resource to understand the nature and scale of impact as well as the key drivers that help ensure a move from ‘bench to bedside’ in biomedical and health research. It is important, however, to acknowledge that these case studies were written for assessment, rather than analytical, purposes, which means further work is needed to extract quantitative information and metrics.

## Making sense of impact data using text mining techniques

In March, we published a report that characterised the 6,679 non-redacted case studies submitted to REF 2014 [5], of which 1,594 (24 %) were in Panel A, which included biomedicine, health, and clinical disciplines. To trawl through the large amount of text available in the case studies (there were more than 6 million words in ‘details of the impact’ sections alone) text mining techniques were used in three ways: i) topic modelling was used to uncover hidden thematic structures or ‘topics’ that occurred in the documents, ii) keyword searches were used to look for specific instances of impact (e.g., Quality Adjusted Life Years (QALYs)) or specific organisations (e.g., NICE), and iii) information extraction was used to match third party information with the case studies, typically around proper nouns such as countries, cities, and institutions.

The analysis of the case studies led to the identification of 60 impact topics or areas where research influences society, such as medical ethics, climate change, clinical guidance, and women, gender, and minorities. In supplementary ‘deep mines’, we read more than 1,000 case studies to provide a deeper picture of the data – and looked at specific questions such as ‘what is the impact and value of research on clinical practice and health gain?’ and ‘what has been the impact of research on BRIC countries?’

## UK HEI research is multidisciplinary, multi-impactful, and multinational

One of the most striking observations from the analysis is the diverse range of contributions that UK HEIs have made to society, ranging from improvements in access to care for tuberculosis patients in African countries to the development of a super-repellent surface invented by UK researchers now used in products worldwide such as mobile phones and hearing aids. Parallel to this is the largely multidisciplinary nature of the research that underpins the impacts identified. The analysis has shown that a very large proportion of the case studies draw on underpinning research from diverse disciplines [6]. To illustrate this, 156 fields of research within 36 Units of Assessment were linked to the 60 impact topics resulting in 3,709 unique ‘pathways to impact’.

## Estimating health gains

Beyond the general themes and patterns identified in the data, any further quantitative analysis is challenging to automate from the case studies; in the ‘details of the impact’ sections of the case studies, approximately 70,000 pieces of numerical information, excluding dates, were identified. The possibility of monetising the health gain from UK HEI research by qualitatively extracting information in the case studies was explored. One approach for calculating the health gain from an intervention is to use QALYs, where one QALY is a measure of the health gain from a treatment equivalent to 1 year of perfect health. A keyword search on the term “QALY” identified 23 case studies (all but one from Panel A). Reading these case studies determined that for 12 of them, the QALY was being used to illustrate the cost-effectiveness of an intervention, but for the remaining 11, researchers evidenced and monetise the actual or potential health gain arising from the underpinning research in the case studies. These latter case studies were used to crudely estimate a potential net total gain of around £2 billion in the impact period 2008 to 2012, although we stress this is an indicative estimate.

To obtain this figure, the monetary benefit from the data given in the case studies was calculated, but data had to be supplemented and manipulated in various ways. For example, the information presented in the case studies was neither consistent nor standardised, with some case studies presenting the QALY gain for an individual patient, whereas others for a patient population. Some case studies provided an estimate of the net monetary benefit, but different figures for the value of a QALY (ranging from £25,000 to £40,000) were used. In addition, for some of the case studies, additional information had to be sourced from the cited material and, in one case, an external source was referred to that was not cited in the case study (as it was not published until after the case study had been submitted).

## Looking ahead

There are a number of key lessons arising from this research. Whilst an invaluable resource, there are limitations to using the case studies for analytical purposes. The case studies were written for assessment purposes, which meant there was often a universally positive sentiment in the language used, researchers could carefully select which case studies were included, and there was a small number of identical or near-identical submissions. Furthermore, while some quantitative data is available in the case study narratives, there was no requirement or mechanism for standardised reporting of metrics or impact outputs. As shown in the health gain example, producing return on investment type figures requires further work in matching the data available to external data sources. The key message here is that it is possible to extract valuable information and patterns on impact arising from UK HEI research. In order to optimise this process, however, these case studies would need to be re-purposed from an advocacy tool to an analytical one. By improving how we analyse and report non-academic impact of HEI research, we can begin to understand what works in research funding, research translation, and the broader research system.
